# Characterization of *Mycobacterium tuberculosis*–Specific Th22 Cells and the Effect of Tuberculosis Disease and HIV Coinfection

**DOI:** 10.4049/jimmunol.2200140

**Published:** 2022-08-01

**Authors:** Mohau S. Makatsa, F. Millicent A. Omondi, Rubina Bunjun, Robert J. Wilkinson, Catherine Riou, Wendy A. Burgers

**Affiliations:** * Division of Medical Virology, Department of Pathology, Faculty of Health Sciences, University of Cape Town, Cape Town, South Africa;; † Wellcome Centre for Infectious Diseases Research in Africa, University of Cape Town, Cape Town, South Africa;; ‡ Institute of Infectious Disease and Molecular Medicine, University of Cape Town, Cape Town, South Africa;; § Department of Medicine, Imperial College London, London, U.K.; and; ¶ Francis Crick Institute Mill Hill laboratory, London, U.K.

## Abstract

The development of a highly effective tuberculosis (TB) vaccine is likely dependent on our understanding of what constitutes a protective immune response to TB. Accumulating evidence suggests that CD4^+^ T cells producing IL-22, a distinct subset termed “Th22” cells, may contribute to protective immunity to TB. Thus, we characterized *Mycobacterium tuberculosis*–specific Th22 (and Th1 and Th17) cells in 72 people with latent TB infection or TB disease, with and without HIV-1 infection. We investigated the functional properties (IFN-γ, IL-22, and IL-17 production), memory differentiation (CD45RA, CD27, and CCR7), and activation profile (HLA-DR) of *M. tuberculosis*–specific CD4^+^ T cells. In HIV-uninfected individuals with latent TB infection, we detected abundant circulating IFN-γ–producing CD4^+^ T cells (median, 0.93%) and IL-22–producing CD4^+^ T cells (median, 0.46%) in response to *M. tuberculosis*. The frequency of IL-17–producing CD4^+^ T cells was much lower, at a median of 0.06%. Consistent with previous studies, IL-22 was produced by a distinct subset of CD4^+^ T cells and not coexpressed with IL-17. *M. tuberculosis*–specific IL-22 responses were markedly reduced (median, 0.08%) in individuals with TB disease and HIV coinfection compared with IFN-γ responses. *M. tuberculosis*–specific Th22 cells exhibited a distinct memory and activation phenotype compared with Th1 and Th17 cells. Furthermore, *M. tuberculosis*–specific IL-22 was produced by conventional CD4^+^ T cells that required TCR engagement. In conclusion, we confirm that Th22 cells are a component of the human immune response to TB. Depletion of *M. tuberculosis*–specific Th22 cells during HIV coinfection may contribute to increased risk of TB disease.

## Key Points

- IL-22 is produced by conventional CD4+ T cells that require TCR engagement.
- Th22 cells are markedly reduced during TB and HIV coinfection compared to Th1 cells.
- Th22 cells exhibit shared and distinct features compared to Th1 and Th17 cells.

## Introduction

Tuberculosis (TB) is the leading cause of death from an infectious disease worldwide, with 10 million TB cases per year and 1.6 million deaths in 2018 (1). This study was conducted in South Africa, which is one of the eight countries that together account for two thirds of all TB cases globally (1). Moreover, approximately one quarter of the world’s population is latently infected with *Mycobacterium tuberculosis* (1). In immunocompetent individuals, the risk of progression from infection to active TB disease is 2–10% in a lifetime, illustrating that the human immune system can control *M. tuberculosis* in most cases (2). Bacillus Calmette–Guérin (BCG) is the only licensed TB vaccine and protects against disseminated TB in children but provides variable protection against highly prevalent pulmonary TB in adults (3, 4). Therefore, there is an urgent need for new and effective TB vaccines, and recent progress in clinical and preclinical trials has delivered promising results (5–7).

A better understanding of the immune responses required to control *M. tuberculosis* will aid the development of improved vaccines against TB (8). It is well established that CD4^+^ T cells, particularly Th1 cells producing the cytokines IFN-γ and TNF-α, are critical for immunity against *M. tuberculosis* (8, 9). However, Th1 immunity alone is not sufficient, as IFN-γ has been reported to be a poor correlate of BCG vaccination-induced protection against TB in mice (10, 11). Furthermore, IFN-γ–independent mechanisms of CD4^+^ T cell–mediated control of *M. tuberculosis* infection have been documented (12–14). Thus, other CD4^+^ Th subsets beyond Th1 cells may be essential for protection against TB.

There is growing interest in the cytokine IL-22 and its role in TB immunity. IL-22 belongs to the IL-10 family of cytokines, and its receptor is composed of two heterodimeric subunits, IL-22R1 and IL-10R2 (15). IL-22 mainly targets nonhematopoietic cells, namely epithelial cells, and fibroblasts in tissues (15), but expression of the IL-22 receptor has also been reported on macrophages (16, 17). IL-22 promotes tissue proliferation, regeneration, and healing (15–20). It induces the production of antimicrobial peptides and proteins such as β-defensins, the S100 family of peptides, Reg3, calprotectin, and calgranulin A (18–20). Furthermore, IL-22 signaling regulates chemokine expression to orchestrate the recruitment of immune cell subsets to sites of infection (20, 21). During infection with *M. tuberculosis*, IL-22 was initially reported to be dispensable for *M. tuberculosis* control in mouse models (22, 23). Recently, however, a protective role for IL-22 in TB immunity was described in a murine model, where IL-22–deficient mice displayed greater bacterial burdens after aerosol infection with a virulent clinical strain of *M. tuberculosis*, HN878 (17). In humans, soluble IL-22 has been detected at sites of extrapulmonary TB (24), and a higher concentration of IL-22 was observed in bronchoalveolar lavage fluid of individuals with active TB compared with healthy donors (25). Moreover, IL-22 has been shown to inhibit intracellular *M. tuberculosis* growth in macrophages (16), and a polymorphism in the IL-22 promoter has been linked to increased TB risk (26).

IL-22 is produced by a variety of cells, including T cells (Th17, Th1 and γδ T cells) and innate cells (innate lymphoid cells and NK cells) (16, 27, 28). In humans, IL-22 is mainly produced by a distinct subset of CD4^+^ T cells, named Th22 cells (29, 30). Our laboratory recently showed that IL-22–producing CD4^+^ T cells contribute to the mycobacterial response during latent TB infection (LTBI) (31). These mycobacteria-specific Th22 cells were depleted during HIV infection to a similar extent as Th1 cells, emphasizing their potential importance in protective immunity to TB in the context of HIV coinfection. In the current study, we further characterized *M. tuberculosis*–specific Th22 cells during TB disease and HIV coinfection. This study investigated the contribution of Th22 cells to TB immunity, characterized Th22 cells further to gain insights into their function, and examined the impact of TB disease and HIV infection on Th22 cells, in comparison with Th1 and Th17 cells. Our findings confirm that IL-22 is produced by CD4^+^ T cells in response to *M. tuberculosis* Ags, and these Th22 cells contribute substantially to *M. tuberculosis* immune responses. Moreover, *M. tuberculosis*–specific Th22 cells were severely diminished in patients with both HIV infection and TB disease. Additionally, we demonstrate, to our knowledge, for the first time, that IL-22 production is dependent on TCR engagement but may require alternative costimulatory molecules or APCs.

## Materials and Methods

### Study participants

Blood samples were collected from 72 individuals recruited from the Ubuntu Clinic, Khayelitsha, Cape Town, South Africa. Participants were classified into four groups according to their HIV-1 infection status and whether they had LTBI or active TB disease (aTB): HIV^−^/LTBI (*n* = 19), HIV^+^/LTBI (*n* = 18), HIV^−^/aTB (*n* = 19), and HIV^+^/aTB (*n* = 16). The clinical characteristics for each group are presented in Table I. LTBI was diagnosed based on a positive IFN-γ release assay (QuantiFERON-TB Gold In-Tube test), no symptoms of aTB, and no detection of *M. tuberculosis* in sputum by GeneXpert. Diagnosis of aTB was based on clinical symptoms and a positive sputum test (GeneXpert). All HIV-infected individuals were antiretroviral treatment-naïve, and all TB cases were drug-sensitive and TB treatment-naive at the time of enrolment. All participants had received BCG vaccination at birth. In addition to the patient cohort, healthy adult donors were recruited from the University of Cape Town for selected experiments. All participants gave written, informed consent. These studies were approved by the University of Cape Town, Faculty of Health Sciences Human Research Ethics Committee (HREC 279/2012 and HREC 896/2014).

**Table I.** Clinical characteristics of the four study groups

	LTBI		Active TB	
HIV^−^	HIV^+^	HIV^−^	HIV^+^
*n*	19	18	19	16
CD4 count (cells/mm^3^)*^a^*	923 (687–1051)	547 (463–626)	648 (593–778)	153 (75–207)
Log_10_ HIV viral load (RNA copies/ml)*^a^*	n.a.	4.61 (3.72–4.97)	n.a.	5.06 (4.68–5.54)
Age (y)*^a^*	25 (19–31)	33 (29–38)	31 (26–40)	34 (29–46)
Sex (female/male)	9/10	16/2	2/17	9/7

n.a., not applicable.

a Median (interquartile range).

### Blood collection and whole blood stimulation

Blood was collected in sodium heparin tubes and processed within 4 h of collection. Whole blood assays were performed according to the protocol described previously (32). Briefly, 0.5 ml of heparinized whole blood was stimulated with *M. tuberculosis* cell lysate (strain H37Rv, 10 μg/ml, BEI Resources), a *M. tuberculosis*–specific peptide pool consisting of 17 and 16 peptides covering the entire ESAT-6 (early secretory antigenic target-6) and CFP-10 (culture filtrate protein-10), respectively (4 μg/ml), BCG (multiplicity of infection of 4, *Mycobacterium bovis* Danish strain 1331, SSI), or gamma-irradiated *M. tuberculosis* whole cells (strain H37Rv, 600 μg/ml, BEI Resources) at 37°C for a total of 12 h in the presence of the costimulatory Abs anti-CD28 and anti-CD49d (1 μg/ml each; BD Biosciences). Unstimulated cells were incubated with costimulatory Abs only. After 7 h, brefeldin A (10 µg/ml; Sigma-Aldrich) was added. RBCs were lysed with an alternative lysing solution (150 mM NH_4_Cl, 10 mM KHCO_3_, 1 mM Na_4_EDTA), and the cell pellet was subsequently stained with LIVE/DEAD Fixable Violet stain (Molecular Probes) or LIVE/DEAD Fixable near-IR stain (Invitrogen). Cells were then fixed using FACS lysing solution (BD Biosciences) and cryopreserved in FCS containing 10% DMSO for batch staining. For lymphocyte-specific protein tyrosine kinase (Lck) inhibition, whole blood from healthy individuals was incubated with increasing concentrations (1–60 μM) of Lck inhibitor (7-cyclopentyl-5-(4-phenoxyphenyl)-7*H*-pyrrolo[2,3-*d*]pyrimidin-4-ylamine, Sigma-Aldrich) for 30 min prior to cell stimulation.

### Intracellular cytokine staining and flow cytometry

To measure the frequency of IL-22–producing cells, cryopreserved cells were thawed, washed, and then permeabilized with Perm/Wash buffer (BD Biosciences). Cells were incubated at 4°C for 1 h with the following Abs: CD19 Pacific Blue (6D5; BioLegend), CD14 Pacific Blue (M5E2; BioLegend), CD3 BV650 (OKT3; BD Biosciences), CD4 PerCP-Cy5.5 (L200; BD Biosciences), CD45RA BV570 (HI100; BioLegend), CD27 PE-Cy5 (1A4CD27; Beckman Coulter), CCR7 PECF594 (3D12; BD Biosciences), HLA-DR allophycocyanin-Cy7 (L243; BD Biosciences), IFN-γ Alexa Fluor 700 (B27; BD Biosciences), IL-17 Alexa Fluor 647 (N49-653; BD Biosciences), and IL-22 PE (22URTI; eBioscience). To investigate which T cells produce IL-22 and IFN-γ, cells were stained with surface Abs (CD3 BV650, CD4 ECD [T4; Beckman Coulter], CD8 QD705 [3B5; Life Technologies], CD56 PE-Cy7 [NCAM 16.2; BD Biosciences], pan γδ PE [IMMU510; Beckman Coulter], CD161 Alexa 647 [HP-3G10; eBioscience], and Vα7.2 BV510 [3C10; BioLegend]) before permeabilization with Perm/Wash buffer (BD Biosciences) and followed by intracellular staining with IFN-γ Alexa Fluor 700 and IL-22 BV421 (22URTI; eBioscience). Stained cells were acquired on a BD LSRFortessa and analyzed using FlowJo (v10, Tree Star). A positive cytokine response was defined as at least twice the background cytokine response from unstimulated cells. To define the phenotype of cytokine-producing cells, a cutoff of 20 cytokine events was used. The gating strategy applied is presented in Supplemental Fig. 1.

### Determining the TCR Vβ repertoire of cytokine^+^ cells

The IOTest Beta Mark TCR Vβ repertoire kit (Beckman Coulter) was used to determine the TCR Vβ repertoire of *M. tuberculosis*–specific IFN-γ– or IL-22–producing CD4^+^ T cells. Briefly, cells from *M. tuberculosis* lysate-stimulated whole blood were stained with CD3 BV650 (OKT3; BD Biosciences), CD4 PerCP-Cy5.5 (L200; BD Biosciences), IFN-γ Alexa Fluor 700 (B27; BD Biosciences), IL-22 allophycocyanin (22JOP; eBioscience), and each of the eight vials containing mixtures of conjugated TCR Vβ Abs corresponding to 24 different specificities.

### Statistical analysis

All statistical tests were performed using Prism (v6; GraphPad Software). Nonparametric tests were used for all comparisons. The Kruskal–Wallis test with Dunn’s multiple comparison test was used for multiple comparisons and the Mann–Whitney *U* test and Wilcoxon matched pairs test were used for unmatched and paired samples, respectively. A *p* value <0.05 was considered statistically significant.

## Results

### Characterization of CD4 Th22 cells in the immune response to *M. tuberculosis* in HIV^−^/LTBI individuals

**FIGURE 1.**
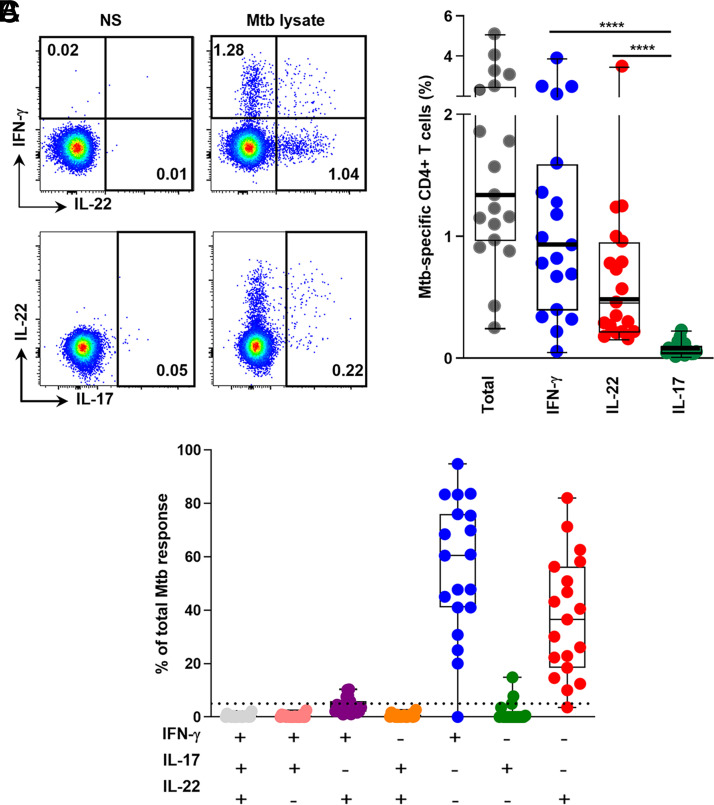
Contribution of IFN-γ, IL-22, and IL-17 to *M. tuberculosis*–specific CD4^+^ T cell responses in individuals with latent TB infection (LTBI). (**A**) Representative flow cytometry plots of IFN-γ, IL-22, and IL-17 production after stimulation with *M. tuberculosis* lysate. NS, no stimulation. (**B**) Summary graph of the frequency of cytokine responses. Statistical comparisons were performed using a one-way ANOVA Friedman test. ****p < 0.0001. (**C**) Proportion of different combinations of IFN-γ, IL-22, and IL-17 in response to *M. tuberculosis* stimulation. Medians and interquartile ranges are depicted. The dotted line represents a 5% threshold.

A subset of CD4^+^ T cells that produces the cytokine IL-22 in response to mycobacterial Ag stimulation has been described (29–31). We sought to further characterize *M. tuberculosis*–specific IL-22 production and define the relative contribution of IL-22 compared with more frequently measured responses, namely IFN-γ and IL-17. We first compared the magnitude of *M. tuberculosis*–specific IL-22–producing CD4^+^ T cells to IFN-γ and IL-17 responses in whole blood from healthy individuals with latent *M. tuberculosis* infection (*n* = 19; (Fig. 1A). *M. tuberculosis*–specific CD4^+^ T cell responses (producing any of the measured cytokines) were detected in all individuals (median, 1.34%; interquartile range [IQR], 0.97–2.52). The highest frequency was observed for IFN-γ responses (median, 0.93%; IQR, 0.40–1.60). Although lower, the magnitude of IL-22^+^ *M. tuberculosis*–specific CD4^+^ T cells was not significantly different from the IFN-γ response (median, 0.46%; IQR, 0.22–0.96). In contrast, IL-17–producing CD4^+^ T cells were detectable at much lower frequencies (median, 0.06%; IQR, 0.04–0.11), ∼16- to ∼8-fold lower than IFN-γ (*p* < 0.0001) and IL-22 (*p* = 0.0002) responses, respectively (Fig. 1B). Using a Boolean gating strategy, we next assessed all possible cytokine combinations to determine the coexpression profile of IFN-γ, IL-22, and IL-17 in *M. tuberculosis*–specific CD4^+^ T cells. (Fig. 1C shows that the single IFN-γ response accounted for a median of 60% of the total *M. tuberculosis* response, and single IL-22–producing cells contributed a median of 37% to the *M. tuberculosis* response. Cytokine coexpression was marginal, with only a small proportion of IFN-γ/IL-22 coexpressing cells observed (median, 3.6%; IQR, 2–6). Only 3 out of 19 individuals produced IL-17 alone that contributed >5% to the total *M. tuberculosis* response (Fig. 1C).

**FIGURE 2.**
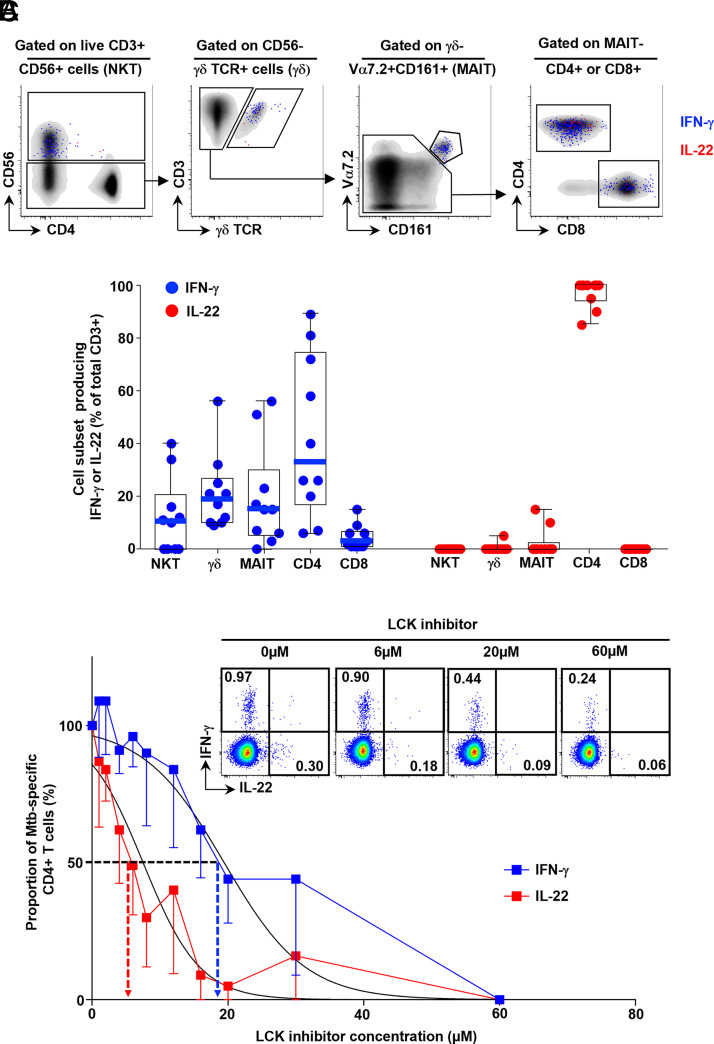
IL-22 responses from different cell populations and the impact of TCR blocking on IFN-γ and IL-22 production. (**A**) Representative flow cytometry plots showing the gating strategy for NKT, γδ T, MAIT, CD4^+^ T, and CD8^+^ T cells. The overlaid dots indicate IFN-γ (blue) or IL-22 (red) production from live CD3^+^ lymphocytes in response to *M. tuberculosis* lysate. (**B**) The frequencies of each subset are indicated as a percentage of total live CD3^+^ cells. IFN-γ (blue) and IL-22 (red) responses to *M. tuberculosis* lysate from NKT, γδ T, MAIT, CD4^+^ T, and CD8^+^ T cells in healthy donors (*n* = 10). Each dot represents one individual. (**C**) Representative flow cytometry plots showing *M. tuberculosis*–specific IFN-γ and IL-22 responses in the presence of different concentrations of the Lck inhibitor 7-cyclopentyl-5-(4-phenoxyphenyl)-7*H*-pyrrolo[2,3-*d*]pyrimidin-4-ylamine and summary graph (*n* = 5). Medians and interquartile ranges are depicted. Vertical arrows show ED_50_. A nonlinear regression curve fit was used.

We next sought to determine whether similar cytokine profiles were generated in response to different types of *M. tuberculosis* Ags, and thus compared blood stimulated with *M. tuberculosis* lysate to gamma-whole cell *M. tuberculosis* (gamma-irradiated) and live mycobacteria (*M. bovis* BCG), as well as *M. tuberculosis* peptides, using an ESAT-6 and CFP-10 peptide pool. No differences in the magnitude of IL-22, IFN-γ, or IL-17 responses were observed between the different “complex” mycobacterial Ags tested (i.e., *M. tuberculosis* cell lysate, gamma-irradiated *M. tuberculosis*, and BCG) using five donors with LTBI (Supplemental Fig. 2A, 2B). This indicates that lysed live or dead mycobacteria can detect IL-22 responses. In contrast, IL-22 production was barely detectable in response to *M. tuberculosis* peptide stimulation in 11 donors with LTBI (Supplemental Fig. 2C). These data raised two questions: 1) does IL-22 production originate from unconventional T cells or 2) is IL-22 production induced by a TCR-independent pathway (33)? A variety of immune cells have the ability to produce IL-22 (34, 35). Thus, to address the first question, we investigated IL-22 and IFN-γ production from a range of T cell subsets in whole blood, including mucosal-associated invariant T (MAIT) cells, γδ T cells, NKT cells, CD4^+^ T cells, and CD8^+^ T cells. Samples were gated on live lymphocytes that were CD3^+^, and then defined as NKT (CD56^+^), γδ T (CD56^−^γδTCR^+^), MAIT (CD56^−^γδTCR^−^Vα7.2^+^CD161^+^), CD4 T (NKT^−^γδTCR^−^MAIT^−^CD8^−^CD4^+^), and CD8 T (NKT^−^γδTCR^−^MAIT^−^CD4^−^CD8^+^) cells (Fig. 2A). In 10 healthy donors tested for cytokine responses, all cell subsets of interest produced IFN-γ when stimulated with *M. tuberculosis* lysate. In contrast, in 7 out of 10 donors, IL-22 was exclusively produced by CD4^+^ T cells, with only 3 donors demonstrating a minor contribution of either MAIT or γδ T cells to the total IL-22 response from CD3^+^ cells (Fig. 2B). Of note, most MAIT and γδ T cells were CD4^−^ (data not shown), indicating that our gating strategy used in the previous experiments was not likely to have included IL-22 from these T cell sources. Thus, conventional CD3^+^CD4^+^ T cells appear to be the major source of IL-22 in response to *M. tuberculosis* Ags.

To determine whether IL-22 production was *M. tuberculosis* specific (i.e., stimulated via recognition of cognate Ag by the TCR) or due to a bystander effect of cytokine activation of nonspecific cells, we inhibited the TCR pathway by blocking Lck (a tyrosine kinase critical for early propagation of TCR signaling) using increasing concentrations of the Lck inhibitor 7-cyclopentyl-5-(4-phenoxyphenyl)-7*H*-pyrrolo[2,3-*d*]pyrimidin-4-ylamine. Using whole blood from five donors with LTBI, we found that as for IFN-γ, IL-22 production was suppressed upon Lck inhibition in a dose-dependent manner. Interestingly, the ED_50_ for IL-22 inhibition was 2.6-fold lower compared with that of IFN-γ inhibition (7.5 and 19.5 µM, respectively; (Fig. 2C). These data demonstrate that IL-22 production from CD4^+^ T cells is TCR-dependent.

**FIGURE 3.**
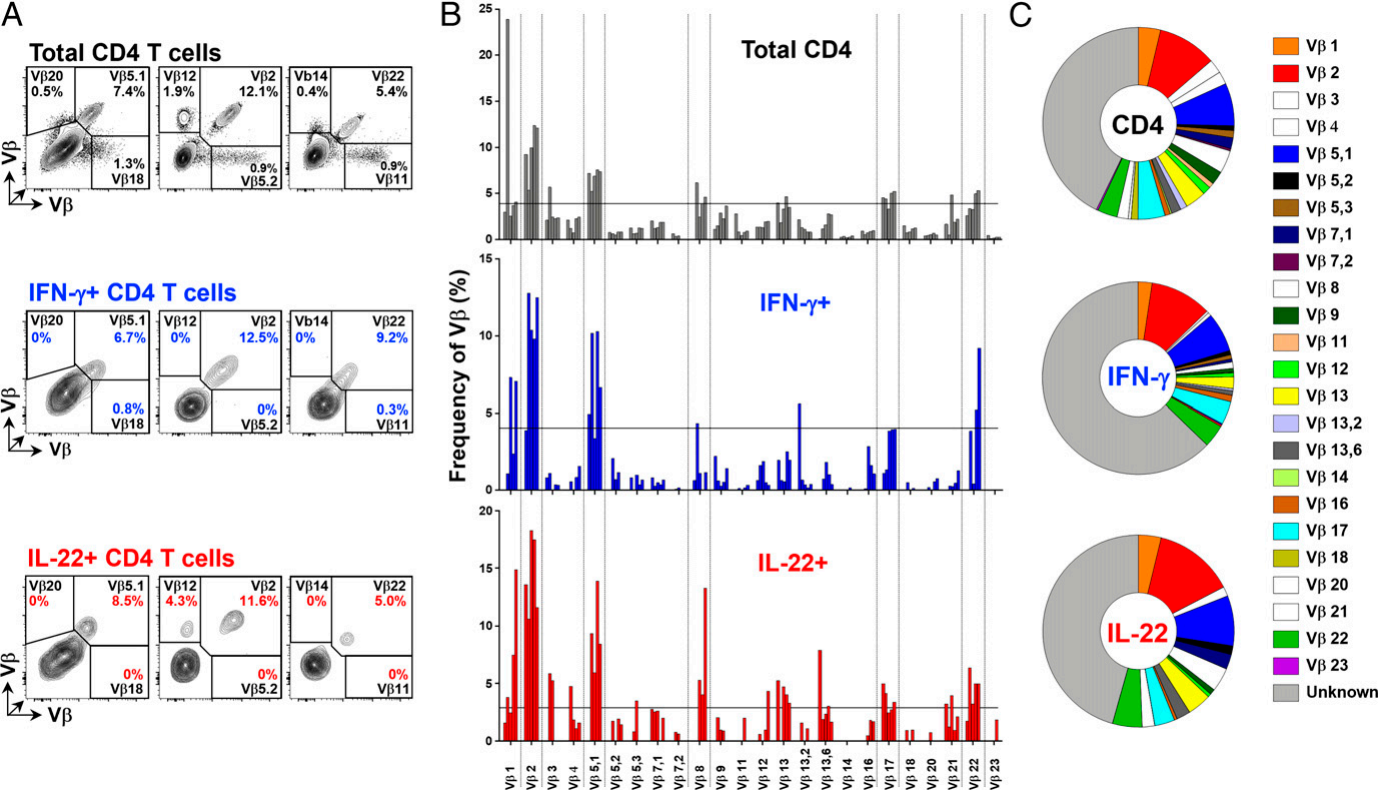
TCR Vβ repertoire of IFN-γ and IL-22–producing CD4^+^ T cells. (**A**) Representative flow cytometry plots showing staining of 9/24 Vβ receptors on total CD4^+^ T cells and IFN-γ– and IL-22–producing CD4^+^ T cells. (**B**) Bar graph (each bar represents a donor) and (**C**) summary pie chart showing expression of 24 Vβ receptors by total CD4^+^ T cells and IFN-γ– and IL-22–producing CD4^+^ T cells (*n* = 5). Means are depicted.

Lastly, we compared the TCRβ repertoire of IL-22 and IFN-γ–producing CD4^+^ T cells in blood from five donors with LTBI, using a commercial typing test. Of note, the kit detects 24 out of 64 known TCR Vβs, representing ∼70% of the overall human TCR Vβ repertoire, as specified by the manufacturer (Beckman Coulter). (Fig. 3A shows representative flow cytometry plots of 9 out of 24 TCR Vβs measured. Vβ repertoire coverage for *M. tuberculosis*–specific IL-22–producing CD4^+^ T cells was broad and comparable to total CD4^+^ T cells, whereas slightly lower coverage was observed for *M. tuberculosis*–specific IFN-γ–producing CD4^+^ T cells (medians 62, 61, and 40%, respectively). Two TCRs, Vβ2 and Vβ5.1, accounted for >5% of the total Vβ repertoire for both *M. tuberculosis*–specific IL-22– and IFN-γ–producing CD4^+^ T cells (median, 13.6 and 10.4% for Vβ2, 8.5 and 6.7% for Vβ5.1, respectively) and were also the most prevalent vβs observed for total CD4^+^ T cells (median, 10.0% for Vβ2 and 7.2% for Vβ5.1, (Fig. 3B). Thus, TCR Vβ repertoire usage was similar between IL-22– and IFN-γ–producing CD4^+^ T cells.

Overall, these results show that IL-22 contributes to a sizeable portion of the *M. tuberculosis* response and that these cells constitute a subset distinct from Th1 or Th17 cells. Moreover, *M. tuberculosis*–specific IL-22–producing CD4^+^ T cells appear to be conventional CD4^+^ T cells and are dependent on TCR signaling for cytokine production.

### *M. tuberculosis–*specific Th22 cells exhibit distinct memory and activation profiles compared with Th1 and Th17 cells

**FIGURE 4.**
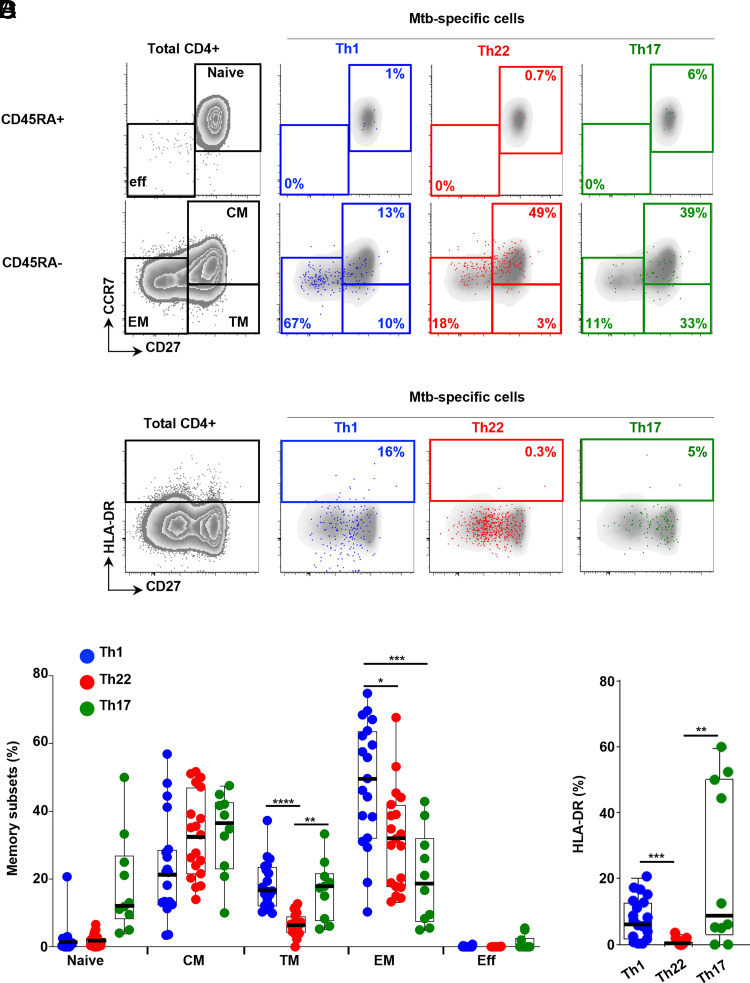
Comparison of the memory differentiation and activation profile of *M. tuberculosis*–specific Th1, Th22, and Th17 cells in LTBI individuals. (**A**) Representative overlay plots showing memory subsets in total CD4^+^ T cells (gray), IFN-γ^+^ (blue), IL-22^+^ (red), and IL-17^+^ (green) cells in response to *M. tuberculosis* lysate (naive, CD45RA^+^CD27^+^CCR7^+^; central memory [CM], CD45RA^−^CD27^+^CCR7^+^; transitional memory [TM], CD45RA^−^CD27^+^CCR7^−^; effector memory (EM), CD45RA^−^CD27^−^CCR7^−^; and effector [Eff] cells, CD45RA^+^CD27^−^CCR7^−^). (**B**) Representative overlay plots of HLA-DR expression in total CD4^+^ T cells (gray), IFN-γ^+^ (blue), IL-22^+^ (red) and IL-17^+^ (green) cells in response to *M. tuberculosis* lysate. (**C** and **D**) Summary graph (*n* = 19) showing distribution of memory subsets (C) and expression of HLA-DR (D) on IFN-γ–, IL-22–, and IL-17–producing CD4^+^ T cells in individuals with LTBI in the absence of HIV infection. Medians and interquartile ranges are depicted. Statistical comparisons were performed using a one-way ANOVA Kruskal–Wallis test. *p < 0.05, **p < 0.01, ***p < 0.001, ****p < 0.0001.

To further describe the phenotypic characteristics of *M. tuberculosis*–specific Th22 cells and compare them to Th1 and Th17 subsets, we defined their memory (CD45RA, CD27, and CCR7) and activation profile (HLA-DR) in healthy individuals with latent *M. tuberculosis* infection. We focused our analysis on IFN-γ single producing cells (Th1), IL-22 single producing cells (Th22), and IL-17 single producing cells (Th17), as the proportion of cytokine coexpressing cells was negligible (Fig. 1C). The measurement of CD45RA, CD27, and CCR7 enabled the detection of five distinct memory subsets, namely naive (CD45RA^+^CD27^+^CCR7^+^), central memory (CD45RA^−^CD27^+^CCR7^+^), transitional memory (CD45RA^−^CD27^+^CCR7^−^), effector memory (EM; CD45RA^−^CD27^−^CCR7^−^), and effector cells (CD45RA^+^CD27^−^CCR7^−^) (Fig. 4A). The memory profile of *M. tuberculosis*–specific CD4^+^ T cells varied depending on their Th polarization. Th1 cells were significantly enriched in the EM phenotype compared with Th22 and Th17 cells (median, 50, 32, and 19%, respectively). Moreover, the Th22 subset was characterized by a low proportion of cells exhibiting a transitional memory phenotype compared with the Th1 or Th17 subsets (median, 6, 17, and 18%, respectively; (Fig. 4C).

When assessing the activation status of *M. tuberculosis*–specific CD4^+^ Th subsets (Fig. 4B), we observed that Th22 cells were characterized by a significantly lower expression of HLA-DR compared with both Th1 and Th17 cells (median of 1.1%, compared with 7.3 and 9.3%, respectively; (Fig. 4D), highlighting that Th22 cells display a different activation status compared with Th17 and Th1 cells. The slightly increased frequency of HLA-DR–expressing Th22 cells observed during HIV infection, unlike Th1 cells, merely mirrored background HIV-induced activation observed on total CD4^+^ T cells. It is plausible that other activation markers may be expressed on activated Th22 cells, and we have observed CD69 and CD25 upregulation on Th22 cells upon *M. tuberculosis* stimulation of blood from latently infected individuals (J.W. Milimu, R. Keeton, and W.A. Burgers, unpublished observations).

Overall, these results indicate that *M. tuberculosis*–specific Th22 cells exhibit distinct memory and activation profiles compared with Th1 and Th17 cells.

### HIV infection and TB disease alter the distribution of *M. tuberculosis*–specific CD4^+^ Th subsets

**FIGURE 5.**
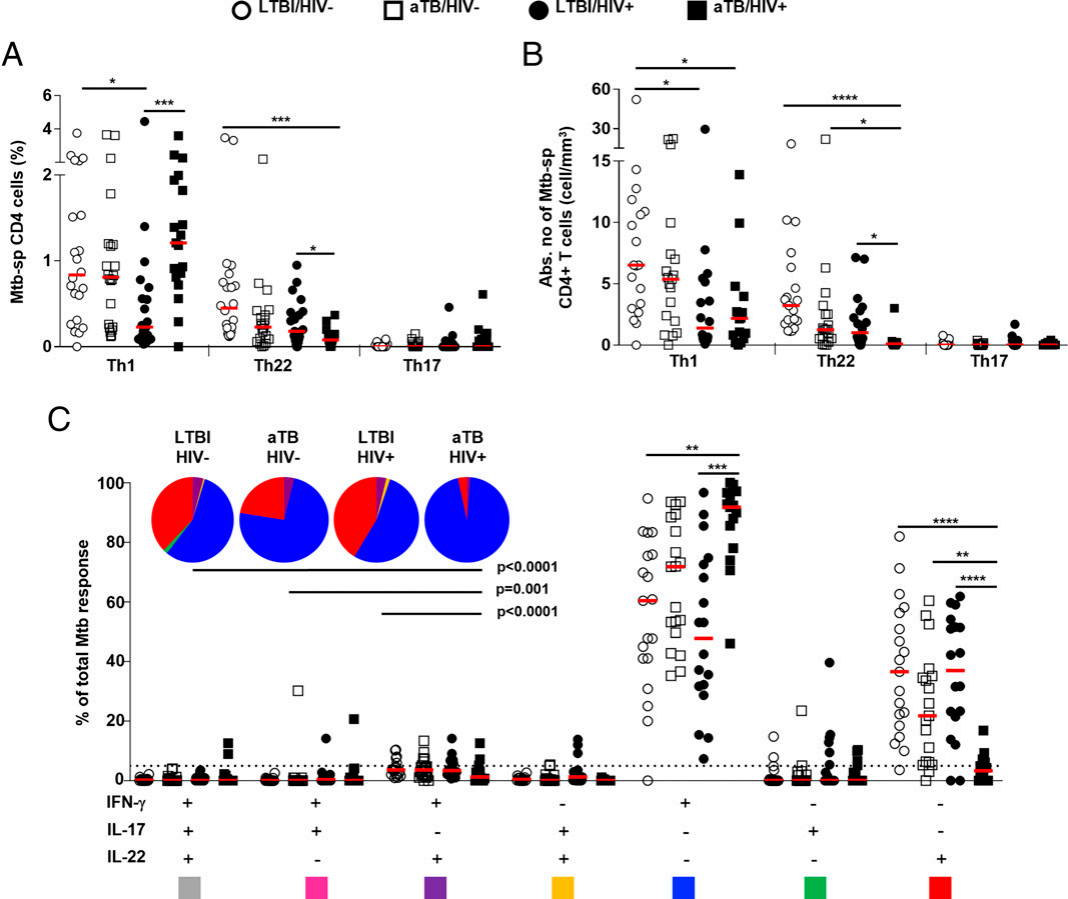
Contribution of IFN-γ, IL-22, and IL-17 to *M. tuberculosis*–specific CD4^+^ T cell responses and the effect of HIV infection and active TB disease. (**A**) Frequency of Th1, Th22, and Th17 responses and (**B**) absolute number of Th1, Th22, and Th17 cells detected in response to *M. tuberculosis* lysate in LTBI/HIV^−^ (*n* = 19), aTB/HIV^−^ (*n* = 19), LTBI/HIV^+^ (*n* = 18), and aTB/HIV^+^ (*n* = 16) individuals. Bars represent the medians. (**C**) Distribution of *M. tuberculosis*–specific cytokine responses. Each section of the pie chart represents median proportions of specific combinations of cytokines, as indicated by the color at the bottom of the graph. The bars represent the median and interquartile range. The dotted line is at 5%. Statistical comparisons were performed using a one-way ANOVA Kruskal–Wallis test. *p < 0.05, **p < 0.01, ***p < 0.001, ****p < 0.0001.

To define the impact of HIV infection and TB disease on the distribution of *M. tuberculosis*–specific Th subsets, we compared the magnitude of Th1, Th22, and Th17 cells in 72 participants classified into four groups according to their HIV-1 and TB status: HIV^−^/LTBI (*n* = 19), HIV^+^/LTBI (*n* = 18), HIV^−^/aTB (*n* = 19), and HIV^+^/aTB (*n* = 16). The clinical characteristics of each group are summarized in Table I. All participants were sampled prior to HIV and/or TB treatment. In participants with LTBI, HIV infection led to a significant reduction (median ∼3.6-fold) in the frequency of *M. tuberculosis*–specific Th1 cells (median, 0.23% for HIV^+^ and 0.84% for HIV^−^; *p* = 0.026; (Fig. 5A). Although not statistically significant, the magnitude of the *M. tuberculosis*–specific Th22 response was also lower in HIV-infected participants compared with HIV-uninfected subjects (median, 0.18 and 0.45%, respectively). When assessing the effect of TB disease, we found that although TB disease did not significantly alter the magnitude of Th1 or Th22 responses in HIV-uninfected individuals, in HIV-infected persons the *M. tuberculosis*–specific Th profile was markedly distorted during TB compared with LTBI (Fig. 5A). Indeed, the magnitude of Th1 responses was significantly higher in aTB/HIV^+^ individuals compared with LTBI/HIV^+^ participants (median, 1.21 versus 0.23%, respectively, *p* = 0.0005). In contrast, *M. tuberculosis*–specific Th22 cells were significantly lower in aTB/HIV^+^ compared with LTBI/HIV^+^ and LTBI/HIV^−^ participants (median, 0.08% versus 0.23 and 0.45%, *p* = 0.046 and *p* < 0.0001, respectively). No differences were observed for *M. tuberculosis*–specific Th17 cells in all four clinical groups.

To account for the significant variation in absolute CD4^+^ T cell counts between groups (Table I), the absolute number of *M. tuberculosis*–specific CD4^+^ T cells was calculated and compared between each group. As expected, HIV coinfection resulted in reduced *M. tuberculosis*–specific Th1 absolute cell numbers regardless of TB status (median, 1.4 versus 6.5 cells/mm^3^ in LTBI and 2.2 versus 5.4 cells/mm^3^ in aTB; (Fig. 5B). A comparable profile was observed for Th22 cells, but due to the decreased frequency of Th22 cells in aTB/HIV^+^ compared with LTBI/HIV^+^ and low CD4 counts in the former group, the absolute number of circulating *M. tuberculosis*–specific Th22 cells was markedly reduced in aTB/HIV^+^ individuals compared with LTBI (median, 0.09 versus 1.02 cells/mm^3^, respectively, (Fig. 5B). Of note, for HIV-infected individuals, we found no relationship between the frequency of any of the Th cytokine responses with absolute CD4 count or HIV viral load, regardless of TB status (data not shown).

Next, we examined how HIV infection and active TB might alter the relative contribution of Th subsets to the total *M. tuberculosis* response. Although there were no significant differences in the contribution of Th1 and Th22 cells to the *M. tuberculosis* response in LTBI/HIV^−^, LTBI/HIV^+^, and aTB/HIV^−^ groups (medians for Th1, 60, 48, and 72%, and medians for Th22, 37, 37, and 22%, respectively), in the aTB/HIV^+^ group, the *M. tuberculosis*–specific response consisted almost exclusively of Th1 cells (>90%), with Th22 cells representing <5% of the total *M. tuberculosis* response (Fig. 5C).

### TB disease and HIV coinfection differentially influence memory and activation profiles of *M. tuberculosis*–specific CD4^+^ Th subsets

**FIGURE 6.**
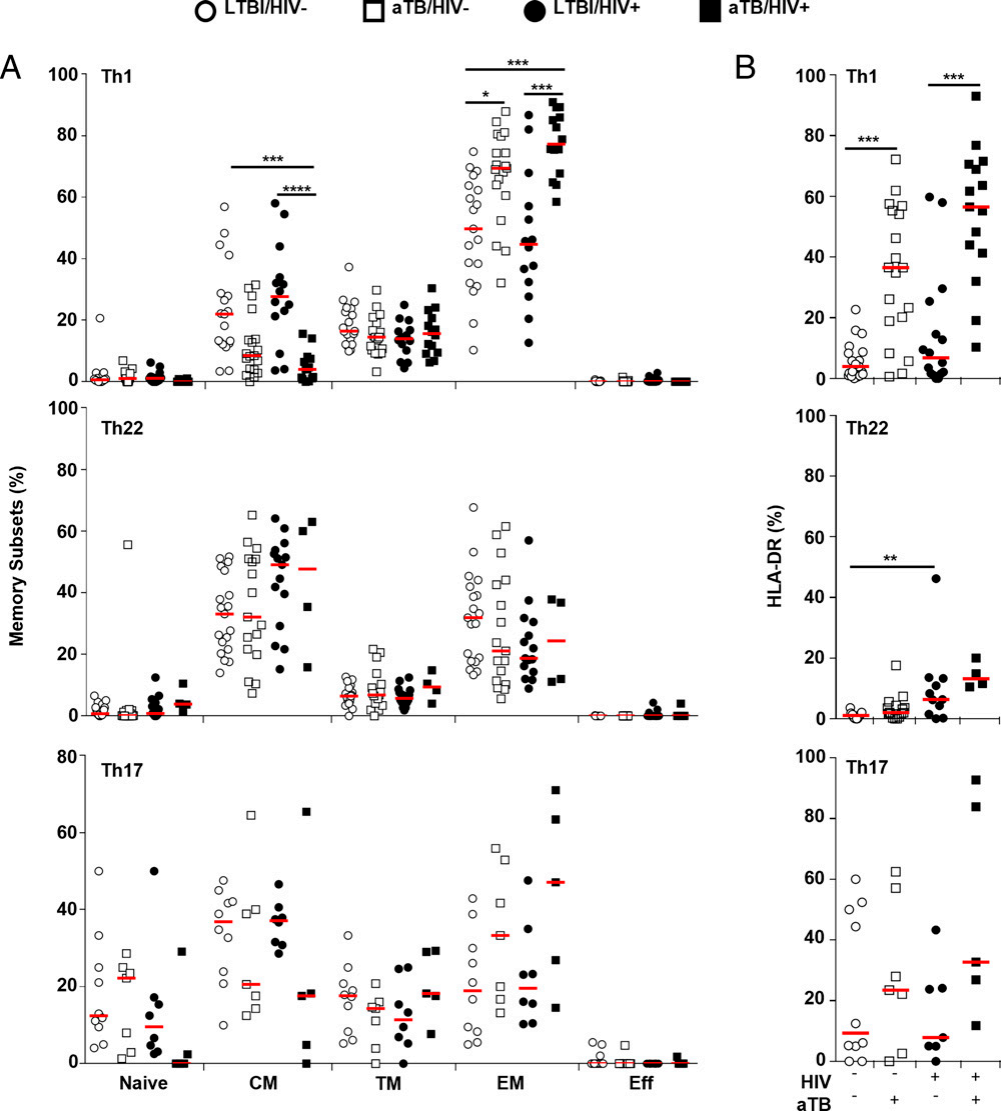
Memory and activation profile of *M. tuberculosis*–specific CD4^+^ T cells in individuals with distinct HIV and TB disease status. (**A**) Memory phenotype and (**B**) HLA-DR expression of IFN-γ–, IL-22–, and IL-17–producing CD4^+^ T cells in LTBI/HIV^−^, aTB/HIV^−^, LTBI/HIV^+^, and aTB/HIV^+^ groups. Red bars represent the medians. Statistical comparisons were performed using a one-way ANOVA Kruskal–Wallis test. *p < 0.05, **p < 0.01, ***p < 0.001, ****p < 0.0001.

Lastly, we performed phenotypic characterization of *M. tuberculosis*–specific CD4 Th subsets during HIV infection and TB disease. Comparing first the memory differentiation phenotype of Th subsets, we showed that TB disease, characterized by active bacterial replication, irrespective of HIV coinfection, promotes the differentiation of *M. tuberculosis*–specific Th1 cells. The proportion of Th1 cells exhibiting an EM phenotype was significantly higher in aTB compared with LTBI (69 versus 50% for HIV^−^, *p* = 0.0295, and 77 versus 45% for HIV^+^, *p* = 0.0004; (Fig. 6A). In contrast, no alteration of the memory differentiation phenotype of Th22 or Th17 cells was observed in aTB. We next assessed the effect of HIV infection and aTB on the activation profile of *M. tuberculosis*–specific CD4^+^ Th subsets. As previously described (36), irrespective of HIV infection, *M. tuberculosis*–specific Th1 cells in aTB were characterized by significantly higher expression of HLA-DR when compared with persons with LTBI (median, 45 versus 7%, respectively, for HIV^−^ [*p* = 0.0004], and 60 versus 8% for HIV^+^ [*p* = 0.0009]). However, aTB did not induce any significant changes in HLA-DR expression in Th22 or Th17 cells (Fig. 6B).

These data confirm that *M. tuberculosis* replication during TB disease influences the memory differentiation and activation of *M. tuberculosis*–specific Th1 cells. In contrast, TB disease does not alter the memory or activation profile of *M. tuberculosis*–specific Th22 cells, indicating that TB differentially modulates *M. tuberculosis*–specific Th1 and Th22 cells.

## Discussion

The importance of CD4^+^ Th1 (IFN-γ) and Th17 (IL-17) responses in protective immunity to *M. tuberculosis* is well established (9, 37). The emerging role of IL-22 and Th22 cells in TB immunity is less well studied, and we sought to address this knowledge gap. To better understand the contribution of Th22 cells to *M. tuberculosis* immune responses, we examined the dynamics of this subset in the context of TB disease and/or HIV infection by comparing the magnitude, differentiation, and activation profiles of CD4^+^ T cells producing IFN-γ, IL-22, and IL-17. We confirm and extend our previous observations from latent *M. tuberculosis* infection (31), demonstrating that distinct, *M. tuberculosis*–specific IL-22–producing CD4^+^ T cells contribute a substantial portion to the total CD4^+^ T cell response to *M. tuberculosis* in both latent infection and TB disease. Furthermore, Th22 cells display different memory and activation profiles compared with Th1 and Th17 cells, and *M. tuberculosis*–specific Th22 cells are severely reduced in blood during TB disease in the context of HIV coinfection.

We show that *M. tuberculosis*–specific Th22 cells make up nearly 40% of the total *M. tuberculosis* response measured in both LTBI and in TB disease, with Th1 cells contributing 60% and Th17 cells <3% to the response. In some individuals, the Th22 response was 60–70% of the total CD4^+^ T cell response measured. These data suggest that Th22 cells may contribute to TB immunity. Indeed, IL-22 was recently found to play a protective role against the hypervirulent clinical strain of *M. tuberculosis*, HN878, in a mouse model (17). In this model, an indirect mechanism of *M. tuberculosis* control was reported, where IL-22 acted on lung epithelial cells to induce secretion of antimicrobial proteins, as well as the induction of chemokines that led to enhanced recruitment of macrophages (17). A direct effector function has also been described: although IL-22 receptors are present primarily on nonhematopoietic cells, there is mounting evidence that they may be expressed on macrophages (16, 17). IL-22 inhibited *M. tuberculosis* growth through induction of TNF-α to activate macrophages and enhance phagolysosomal fusion in infected macrophages through calgranulin A expression (16, 17, 38). In addition to these murine, nonhuman primate, and in vitro models demonstrating a role for IL-22 in *M. tuberculosis* control, a human genetic study described polymorphisms in the promoter region of IL-22 leading to decreased IL-22 production that were associated with greater TB susceptibility (26).

IL-22 has been classified as a Th17 cytokine due to coexpression with IL-17 in mouse studies and several shared functions between the two cytokines (39). Consistent with previous studies (29, 31), our results showed that the vast majority of IL-22 is produced independently of IL-17 (and IFN-γ) in humans. This implies a distinct role for IL-22 in the immune response to *M. tuberculosis* compared with IL-17 (40). Indeed, IL-22 was required for *M. tuberculosis* control at the chronic stage of infection, whereas IL-17 was important in the acute stage of *M. tuberculosis* infection in the same mouse model (17, 41). Although IL-22 and IL-17 have some overlapping functions, such as inducing antimicrobial peptides, regulating chemokine expression, and promoting tissue proliferation and healing (17, 18, 39, 41), IL-22 may also mediate mycobacterial control by inducing TNF-α for macrophage activation (16, 17), a function that is more commonly associated with IFN-γ (42). Further studies are warranted to define the relative contribution and potential synergy of IL-22 with IFN-γ and IL-17 in protective immunity to TB.

In accordance with previous data (25), we show that *M. tuberculosis*–specific Th22 responses in the blood were 50% lower in individuals with TB compared with *M. tuberculosis*–exposed individuals. It is likely that in the context of TB disease, *M. tuberculosis*–specific Th22 cells migrate to the lungs. This conjecture is based on the fact that IL-22–producing cells have been detected in the lungs and granulomas of rhesus monkeys with TB (43, 44), that soluble IL-22 has been found to be elevated at the site of disease during both pulmonary and extrapulmonary TB (24, 25), and also that IL-22–producing cells express CCR6, which has been shown to mediate T cell homing to mucosal tissues (31). Two TB treatment studies further support this hypothesis. Suliman et al. (45) found that patients with LTBI who received isoniazid prophylactic treatment had increased frequencies of BCG-specific IL-22–secreting CD4^+^ T cells compared with pretreatment frequencies. Furthermore, Zhang et al. (46) reported an increase in *M. tuberculosis*–specific soluble IL-22 in blood of TB patients at the completion of their anti-TB treatment, compared with before treatment. These studies suggest that reduction of Ag load at the site of disease led to recirculation of Th22 cells to blood.

Consistent with our recent findings (31), HIV infection resulted in a lower frequency of Th1 and Th22 cells in *M. tuberculosis*–exposed individuals, and also those with active TB. The depletion of Th22 cells could be explained by the fact that most Th22 cells express CCR6 (31), and CD4^+^CCR6^+^ cells display an increased permissiveness to HIV infection (47–49), are enriched in HIV DNA (50), and appear to preferentially support HIV replication (51). Indeed, CD4^+^CCR6^+^ cells have been shown to express high levels of the HIV coreceptors CCR5 and CXCR4, important for viral entry, as well as integrin α_4_β_7_, which has been associated with increased HIV susceptibility (47, 48, 52). Furthermore, CCR6^+^ cells lack the ability to secrete β chemokines, which may protect against HIV in an autocrine manner (52). Thus, in the context of both TB disease and HIV infection, the combined effect of cell depletion and cell migration is the likely cause of the strikingly low IL-22 responses we observed in blood.

A range of innate and adaptive immune cells have been reported to produce IL-22 (16, 26, 27). In our study, we found that the predominant source of T cell–derived IL-22 in response to *M. tuberculosis* Ags was conventional CD4^+^ T cells, excluding MAIT, γδ T, and iNKT cells. Interestingly, whereas IL-22 responses were readily detectable upon stimulation with whole or lysed mycobacteria (i.e., *M. tuberculosis* cell lysate, gamma-irradiated *M. tuberculosis*, and BCG), *M. tuberculosis* peptides were not able to stimulate IL-22 responses, raising the question of whether IL-22 production is induced through bystander effects on T cells from structural components present in these mycobacterial Ag preparations, thus leading to cytokine production. We showed, to our knowledge, for the first time, that IL-22 production is indeed mediated by TCR engagement, as demonstrated by inhibition of TCR signaling and resultant abrogation of cytokine production. Furthermore, IL-22–producing CD4^+^ T cells displayed similar Vβ repertoire usage as IFN-γ–producing CD4^+^ T cells. These data underscore our assertion that these are conventional CD4^+^ T cells; however, although conventional, they may not be classically restricted (i.e., dependent on MHC). The lack of detectable IL-22 in response to peptide stimulation suggests that induction of IL-22 may be via alternative Ag presentation mechanisms and/or additional costimulation requirements (53). One possibility is that IL-22 might be induced by modified peptide or nonpeptide Ags, such as lipopeptides or lipids, and we are currently exploring this hypothesis. There is evidence supporting the production of IL-22 by CD4^+^ T cells recognizing CD1a (54, 55), and both CD1a and CD1c can present lipopeptide Ags to conventional T cells (56). Of note, group 1 CD1-restricted T cells specific for microbial lipid Ags display similar αβ TCR usage to peptide-specific T cells (57). A further possibility, given the demonstrated role of Th22 in HN878 infection (17), is that Th22 cells respond to peptides overrepresented in particular clinical strains or lineages, including drug-resistant isolates (58).

In conclusion, we provide evidence that Th22 cells are a major component of the specific adaptive immune response to *M. tuberculosis* during both infection and disease, and that these cells are reduced in blood during HIV coinfection, building on previous work. We hypothesize that Th22 cells could migrate to the lungs and expand for an effective secondary immune response to provide long-lasting protection against TB. To further investigate the specific role of Th22 cells in TB, it will be of interest to compare the profile of Th22 we observed in blood with the site of disease. The recent outcomes of vaccine candidate M72/AS01E demonstrating 50% efficacy against TB (5) and i.v. BCG vaccination showing sterilizing immunity in nonhuman primates (7) provide an ideal setting to assess the potential protective role of *M. tuberculosis*–specific Th22 cells.

## Supplementary Material

Data Supplement
