# Supplementary material for: Characterization of Mycobacterium tuberculosis–Specific Th22 Cells and the Effect of Tuberculosis Disease and HIV Coinfection
Source: J Immunol. 2022 Aug 1;209(3):446–55. doi: 10.4049/jimmunol.2200140 (PMC9339498; doi:10.4049/jimmunol.2200140)
Supplement: Data Supplement [file JI_2200140.zip › ji_2200140_supplemental_1.pdf]

Supplemental Figure 1.

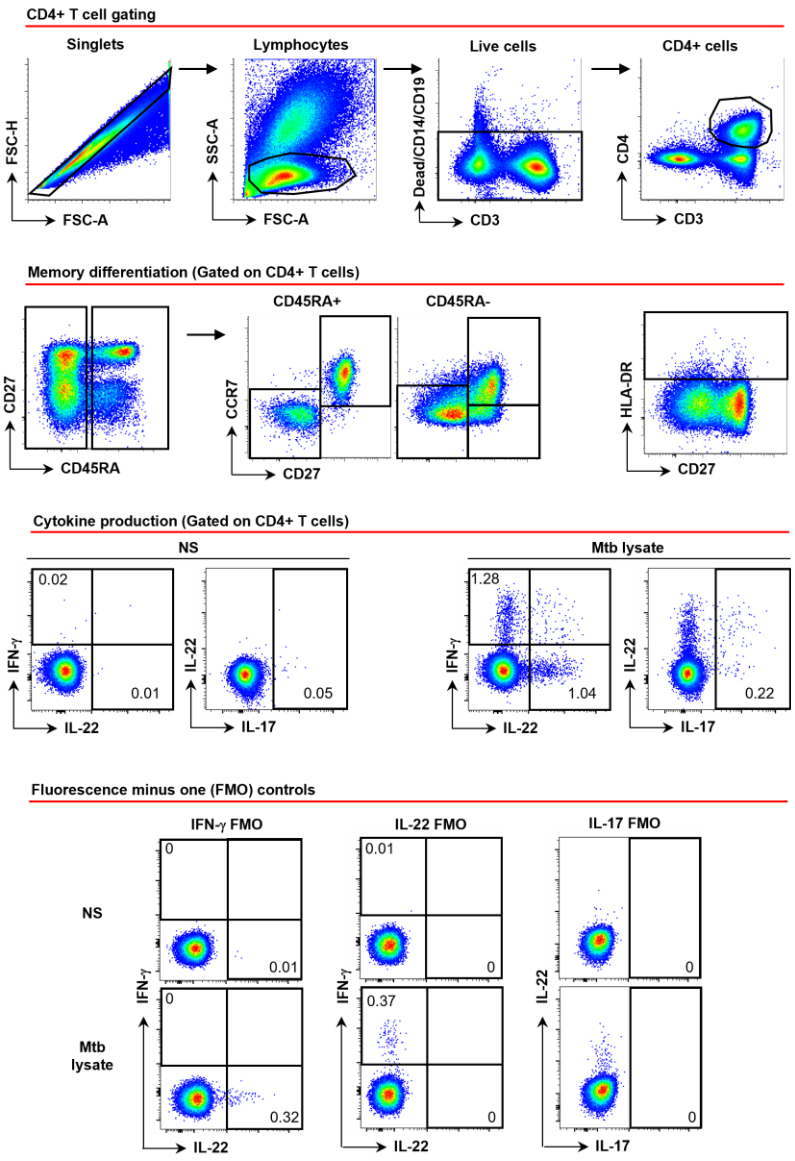

Supplemental Figure S1: Gating strategy. Flow cytometry representative plots indicating gating strategy used to determine the phenotype, memory profile and functional properties of CD4+ T cells and Fluorescent Minus One (FMO) controls for IFN-γ, IL-22 and IL-17 cytokines. NS: non-stimulated, FMO: Fluorescent Minus One.

Supplemental Figure 2.

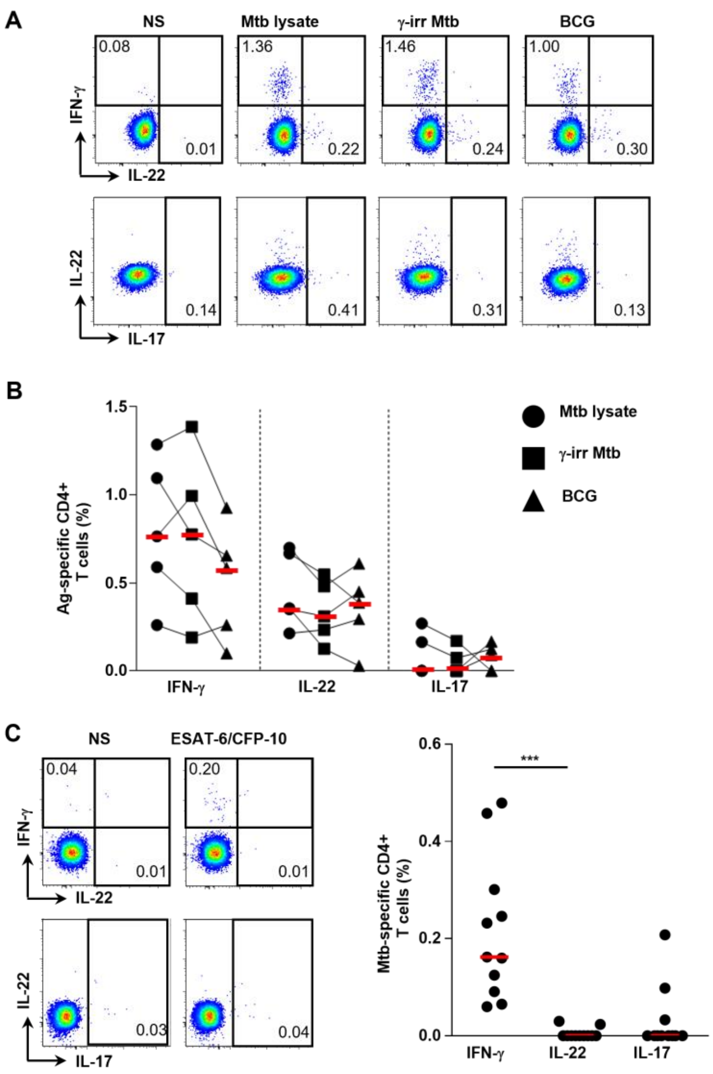

Supplemental Figure S2: Comparison of CD4+ T cell frequencies detected using Mtb lysate, gamma-irradiated Mtb, and BCG. **(A)** Representative flow cytometry plots showing IFN-γ, IL-22 and IL-17 responses after stimulation of blood from healthy donors with Mtb lysate, gamma-irradiated Mtb, and BCG. **(B)** Comparison of the frequency of IFN-γ, IL-22 and IL-17 after stimulation with different antigens (n=5). **(C)** Comparison of the frequency of IFN-γ, IL-22 and IL-17 when stimulated with ESAT-6/CFP-10 (n=11). Red bars represent the medians. Statistical comparisons were performed using a one-way ANOVA Friedman test. *** $p < 0.001$.
